# Anthraquinones with Antiplasmodial Activity from the Roots of *Rennellia elliptica* Korth. (Rubiaceae)

**DOI:** 10.3390/molecules15107218

**Published:** 2010-10-20

**Authors:** Che Puteh Osman, Nor Hadiani Ismail, Rohaya Ahmad, Norizan Ahmat, Khalijah Awang, Faridahanim Mohd Jaafar

**Affiliations:** 1 Faculty of Applied Sciences, Universiti Teknologi MARA, 40450 Shah Alam, Selangor, Malaysia; 2 Centre for Natural Product Research and Drug Discovery (CENAR), Chemistry Department, Faculty of Science, Universiti Malaya, 50603 Kuala Lumpur, Malaysia

**Keywords:** anthraquinone, *Rennellia elliptica*, antiplasmodial, Rubiaceae

## Abstract

Dichloromethane root extract of *Rennellia elliptica* Korth. showed strong inhibition of *Plasmodium falciparum* growth *in vitro* with an IC_50_ value of 4.04 µg/mL. A phytochemical study of the dichloromethane root extract has led to the isolation and characterization of a new anthraquinone, 1,2-dimethoxy-6-methyl-9,10-anthraquinone (**1**), and ten known anthraquinones: 1-hydroxy-2-methoxy-6-methyl-9,10-anthraquinone (**2**), nordamnacanthal (**3**), 2-formyl-3-hydroxy-9,10-anthraquinone (**4**), damnacanthal (**5**), lucidin-*ω*-methyl ether (**6**), 3-hydroxy-2-methyl-9,10-anthraquinone (**7**), rubiadin (**8**), 3-hydroxy-2-methoxy-6-methyl-9,10-anthraquinone (**9**), rubiadin-1-methyl ether (**10**) and 3-hydroxy-2-hydroxymethyl-9,10-anthraquinone (**11**). Structural elucidation of all compounds was accomplished by modern spectroscopic methods, notably 1D and 2D NMR, IR, UV and HREIMS. The new anthraquinone **1**, 2-formyl-3-hydroxy-9,10-anthraquinone (**4**) and 3-hydroxy-2-methyl-9,10-anthraquinone (**7**) possess strong antiplasmodial activity, with IC_50_ values of 1.10, 0.63 and 0.34 µM, respectively.

## 1. Introduction

*Rennellia elliptica* Korth. (Rubiaceae) is a tropical shrub locally known in Malaysia as *Segemuk* or *Mengkudu Rimba*. This plant is widely distributed, along riverbanks or lowland forest, in Peninsular Malaysia, Sumatra and Borneo [1]. Decoctions of the root are taken by the locals for various purposes, including as an aphrodisiac, for body aches and as a post natal tonic [2]. Not much has been reported on the chemical constituents or biological properties of this plant. A preliminary study by Yusoff *et al.* on the roots of the plant reported one anthraquinone compound [3]. Our screening of some Malaysian plant extracts for antiplasmodial activity showed that the dichloromethane roots extract of *R. elliptica* is a potential source of antiplasmodial compounds (IC_50_ = 4.04 µg/mL). This paper reports the isolation, structure elucidation and antiplasmodial activity of a series of anthraquinone compounds, including a new one, from the root of *R. elliptica*. 

**Figure 1:**
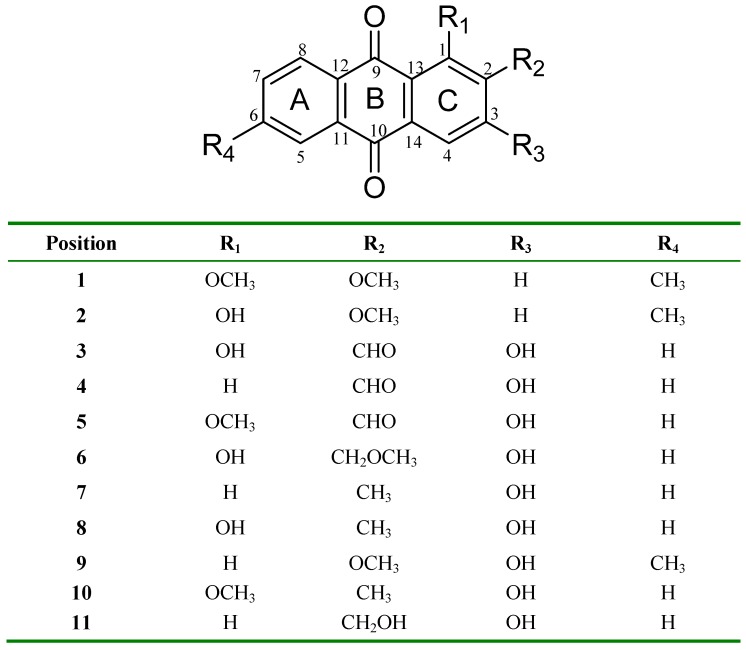
Anthraquinones **1-11**, isolated from roots of *R. elliptica* Korth.

## 2. Results and Discussion

Eleven anthraquinones (Figure 1) were isolated from roots of *R. elliptica* after extensive chromatographic separation and were characterized by analysis of spectroscopic data and by comparison with literature values [4,5,6,7,8,9,10,11,12,13]. The structures of compounds **4** and **6** were also confirmed using x-ray crystallography [14,15]. The compound 1-hydroxy-2-methoxy-6-methyl-9,10-anthraquinone (**2**) was described by Mittie and Biswas in 1928 [16]. However, since then there have been no further reports on the natural occurrence of this compound and there is no spectroscopic data available in the literature for comparison. Hence, we elucidated the structure by careful analysis of MS, IR, UV and NMR data, followed by confirmation through x-ray crystallography [17]. In this paper we include the full spectroscopic data for this compound.

The new compound, 1,2-dimethoxy-6-methyl-9,10-anthraquinone (**1**) was obtained as bright yellow amorphous solid. The HREIMS displayed a [M + H]^+^ peak at 283.0968 [calc 283.3067] suggesting a molecular formula of C_17_H_14_O_4_. The absorption maxima in the UV spectrum were observed at 373, 341 and 257 nm, indicative of an anthraquinone moiety [18]. The IR spectrum did not show presence of chelated carbonyl and hydroxyl groups. The *sp*^2^ C-H stretch for the aromatic ring was observed at 3,081 cm^-1^. With the exception of the sharp singlet in the downfield region for the hydrogen-bonded hydroxyl group, the ^1^H-NMR spectrum resembles that of compound **2**, suggesting a similar substitution pattern. Splitting pattern of the five aromatic proton signals suggested substitutions on both rings. Two overlapping doublets centered at δ_H_ 8.17 are due to H-8 (*d*, *J* = 7.8 Hz) and H-4 (*d, J =* 8.7 Hz), the *peri*-hydrogens. A doublet at δ_H_ 7.28 (*J* = 8.7 Hz) is due to H-3, meanwhile H-7 gave another doublet of doublet at δ_H_ 7.58 (*J*_o_ = 7.8 Hz, *J*_m_ = 1.7 Hz). These assignments were confirmed by their respective correlations in the COSY spectrum. H-5 resonated as a singlet at 8.06 ppm. In addition, two sharp singlets at δ_H_ 2.53 (3H, *s*) and 4.02 (6H, *s*) due to a methyl and two methoxy groups, respectively, were also observed. 

**Figure 2:**
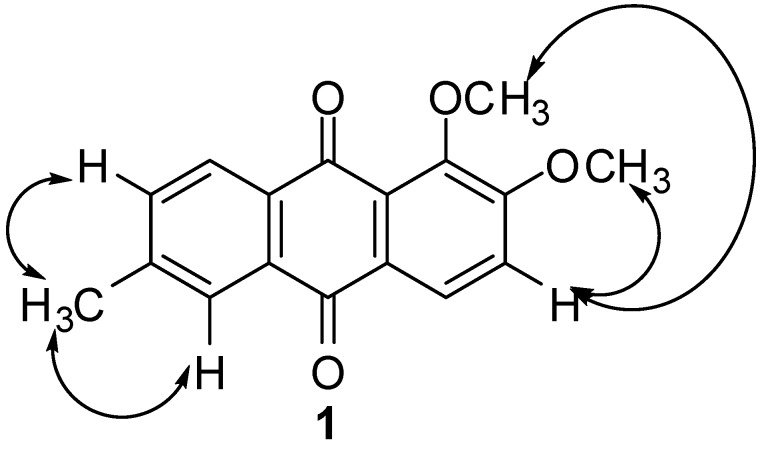
NOESY Correlations of **1**.

The location of the methoxy groups were established at C-1 and C-2 of ring C based on its NOE correlation with H-3. Thus, the only possible location for the methyl substituent is at C-6. This assignment was confirmed through NOE correlations of the methyl group with H-5 and H-7. These NOE correlations are illustrated in Figure 2. The placement of methyl group at C-6 was further confirmed by HMBC experiment which showed a ^3^*J* correlation with H-7. The methine carbons (C-3, C-4, C-5, C-7 and C-8) were assigned through HMQC correlations while the quaternary carbons (C-1, C-2, C-6, C-11, C-12, C-13 and C-14) were assigned based on careful analysis of HMBC spectrum. Both carbonyl carbons in this compound resonated very closely to each other with only 0.01 ppm difference at δ_C_ 182.70 and 182.71, which further confirmed the unchelated nature of the carbonyls. 

Close inspection of all spectroscopic data confirmed that compound **1** is 1,2-dimethoxy-6-methyl-9,10-anthraquinone, the 1-methyl ether of compound **2**. Complete ^1^H and ^13^C data of compounds **1** and **2** are presented in Table 1. 

**Table 1:** ^1^H- (300 MHz) and ^13^C- (75.5 MHz) NMR Data of Compounds **1** and **2** in CDCl_3_.

Position	Compound 1			Compound 2		
	δ_H_	δ_C_	HMBC	δ_H_	δ_C_	HMBC
1	-	159.1	-	-	154.0	-
2	-	149.6	-	-	152.7	-
3	7.28, 1H, *d*, *J* = 8.7 Hz	115.9	C-2, C14	7.19, 1H, *d*, *J* = 8.4 Hz	115.6	C-2, C-14
4	8.17, 1H, *d*, *J* = 8.7 Hz	125.2	C-3, C-10,C-13, C-14	7.89, 1H, *d*, *J* = 8.4 Hz	121.0	C-3, C-10, C-13, C-14
5	8.06, 1H, s	126.9	C-7, C-10, C-11	8.12, 1H, s	127.8	6-CH_3_, C-10, C-11
6	-	144.6	-	-	146.2	-
7	7.58, 1H, *dd*, *J*_o_ = 7.8 Hz, *J*_m_ = 1.7 Hz	134.7	6-CH_3_, C-8, C-12	7.61, 1H, *d*, *J* = 8.1 Hz	134.6	C-6,C-8
8	8.17, 1H, *d*, *J* = 7.8 Hz	127.0	C-6, C-9	8.23, 1H, *d*, *J* = 8.1 Hz	127.1	C-7, C-9, C-12
9	-	182.7	-	-	189.1	-
10	-	182.7	-	-	181.8	-
11	-	132.9	-	-	134.0	-
12	-	132.9	-	-	131.1	-
13	-	127.4	-	-	116.1	-
14	-	127.5	-	-	125.5	-
1-OH	-	-	-	13.20,1H,s	-	
1-OCH_3_	4.02, 3H, s	61.3	C-1	-	-	-
2-OCH_3_	4.02, 3H, s	56.3	C-1,C-2,C-3	4.04, 3H, s	56.4	C-1,C-3
6-CH_3_	2.53, 3H, s	21.8	C-5, C-6, C-7	2.56, 3H, s	22.0	C-5, C-6, C-7

Anthraquinones isolated from the root of *R. elliptica* exhibited the typical substitution pattern of *Rubia* type anthraquinones, with most of them substituted only on ring C [19]. The anthraquinones from *Morinda* and *Prismatomeris* spp. in the same family also exhibited a similar substitution pattern [4,5,6,7,8,9,12,13,20,21,22]. Genus *Rennellia* is classified in the same tribe as *Morinda* and *Prismatomeris* [23], so the anthraquinones may be produced through similar biosynthetic pathways, which explains the similarity in the substitution patterns [19]. However, anthraquinones **1**, **2** and **9** which are substituted on both rings have a methyl substitution at C-6, differing from the anthraquinones of *Prismatomeris* and *Morinda* which are typically hydroxyl or methoxy substituted at C-6. 

We screened the anthraquinones isolated from the roots of *R. elliptica* for antiplasmodial activity based on the promising screening results of dichloromethane crude extract (IC_50_ = 4.04 µg/mL). The *in vitro* antiplasmodial activity of anthraquinones isolated from *R. elliptica* against a chloroquine sensitive strain of *P. falciparum* (3D7) is shown in Table 2. Compound **7** displayed the strongest inhibition activity, with an IC_50_ value of 0.34 µM, followed by compound **4** with an IC_50_ value of 0.63 µM. Sittie *et al.* established that an aldehyde group at C-2 and a phenolic hydroxy group at C-3 on the anthraquinone skeleton enhance the activity of anthraquinones against the growth of *P. falciparum* [24]. Our results show that methyl group at C-2 together with phenolic hydroxy group at C-3 as in compound **7** also gave significant activity. It should also be noted that both compounds **4** and **7** do not possess hydroxyl substituents at the *peri* positions. The new anthraquinone **1** also exhibited strong inhibition, with an IC_50_ value of 1.1 µM. Interestingly, anthraquinone **2**, which structurally differs only at C-1 (hydroxyl substituent instead of methoxy substituent) did not show any significant activity. The position of substituents on anthraquinone skeleton clearly influences the antiplasmodial activity, which warrants further investigation. We are currently synthesizing anthraquinone derivatives with various substitution patterns for the purpose of establishing structure-activity relationships.

**Table 2:** Antimalarial Activities of Anthraquinones from *R. elliptica* Korth.

Sample	IC_50_ (µM)
**1**	1.10
**2**	na^†^
**3**	72.46
**4**	0.63
**5**	51.28
**6**	2.10
**7**	0.34
**8**	na^†^
**9**	nt^‡^
**10**	na^†^
**11**	nt^‡^
**Chloroquine diphosphate**	6.30*

Each sample was tested in duplicate; The IC_50_ values were obtained from average values of percent inhibition within a series of concentration; Notes: na^†^ –no activity; nt^‡^ – not tested; * unit in nM.

## 3. Experimental

### 3.1. Instrumentation

^1^H- and ^13^C-NMR spectra were run on a Bruker 300 Ultrashield NMR (Switzerland) at 300 and 75.5 MHz, respectively, using CDCl_3_ or acetone_-*d*6_ (Merck) as solvent. Chemical shifts are reported in ppm and δ scale with the coupling constants given in Hz. Melting points were determined using a Hinotek X-4 (China) melting point apparatus equipped with a microscope and are uncorrected. HREIMS spectra were obtained on a Thermo Finnigan Automass Multi (Shimadzu, Japan). IR spectra were obtained using Perkin-Elmer 1600 series FTIR spectrometer (USA) using KBr pellets. UV spectra were recorded on Shimadzu UV-160A spectrometer (Japan) in absolute ethanol (Scharlau) and alkaline ethanol. 

### 3.2. Chemicals and Reagents

Column chromatography was carried out using silica gel (silica gel 60, 230-400 mesh, Merck, Germany). *n*-Hexane, dichloromethane and methanol for column chromatography were freshly distilled from industrial grade solvents. The fractions collected were monitored using analytical TLC (Merck, Germany), pre-coated with silica gel 60 F_254_ of 0.25 mm thickness and visualized under UV light at 245 nm and 356 nm. PTLC was carried out using pre-coated plate with PSC-Fertigplatten Kieselgel 60 F_254_ (1.0 mm thickness, 20 × 20 cm) purchased from Merck. *Plasmodium falciparum* (3D7) strain was used for *in vitro* antiplamodial tests. All chemicals used for determination of antiplasmodial activity were purchased from Sigma-Aldrich, unless otherwise stated.

### 3.3. Plant Material

The roots of *R. elliptica* were collected from Kuala Keniam, National Park, Pahang, Malaysia at altitude 165 m above sea level and were identified by Dr Shamsul Khamis, Universiti Putra Malaysia. The voucher specimens (SK1512/08) were deposited at Herbarium of Institute of Bioscience, Universiti Putra Malaysia and Universiti Teknologi MARA.

### 3.4. Extraction and Isolation of Anthraquinones

The powdered air-dried roots of *R. elliptica* (1 kg) were successively extracted with hexane, dichloromethane and methanol (5 L each) at room temperature for 72 hours. The filtrate was concentrated *in vacuo* to give 27 g of a brown coloured residue. The dichloromethane crude extract (9 g) was pre-mixed with silica (1:1) and introduced onto a packed column (5 cm × 60 cm) of acid-washed silica gel (previously shaken with 2% oxalic acid for 2 hours, filtered and dried at 90 ºC). The column was eluted gradiently using compositions of solvents of increasing polarity (*n*-Hex-CH_2_Cl_2_, 9:1, 8:2, 7:3, 6:4, 5:5, 4:6, 3:7, 2:8, 1:9 and 100 CH_2_Cl_2_ v/v; CH_2_Cl_2_-MeOH, 99:1, 95:5, 9:1 v/v). Fractions of 200 mL each were collected and combined based on TLC pattern into eight major fractions (A to H) for further separation procedures.

Compound **3** (92 mg) was obtained upon recrystallization of fraction A from CH_2_Cl_2_. The golden yellow precipitate from fraction C was recrystallized from CH_2_Cl_2_ to yield crystals of **4** (42 mg). The remaining liquid from fraction C was dried and subjected to column chromatography (15 mm × 330 mm) eluted isocratically with CH_2_Cl_2_ to give **2** (7 mg) and **5** (20 mg). Fraction D was purified using PTLC (100% CH_2_Cl_2_) to furnish **6** (23 mg). Compound **7** (20 mg) was purified from fraction E after recrystallization of the dark yellow precipitate from CH_2_Cl_2_. The remaining portion of fraction E was rechromatographed using a small column (15 mm × 330 mm) eluted with a gradient of CH_2_Cl_2_-MeOH (100, 99:1, 98:2, v/v) to give 50 fractions. Repeated PTLC (CH_2_Cl_2_-MeOH, 99:1, v/v) of these fractions afforded compounds **1** (5 mg), **8** (32 mg) and **9** (7 mg). The pale yellow precipitate from fraction F was redissolved in CH_2_Cl_2_ and MeOH. Purification of the MeOH soluble portion using repeated PTLC (CH_2_Cl_2_-MeOH, 97:3, v/v) yielded **10** (23 mg). Compound **11** (5 mg) was obtained upon recrystallization of light yellow precipitate from CH_2_Cl_2_ of fraction F. Spectral data for compounds **3**-**9** are in good agreement with published data [4,5,6,7,8,9,10,11,12,13].

*1,2-Dimethoxy-6-methyl-9,10-anthraquinone* (**1**). Bright yellow amorphous solid (CH_2_Cl_2_); m.p. 193-196 ºC. UV λ_max_ (EtOH): 373, 341, 257 nm; IR ν_max_ (KBr): 3,081, 2,945, 1,666, 1,601 cm^-1^; HREIMS 283.0968 [M + H]^+^ (calc for C_17_H_14_O_4_ 282.3067); For ^1^H-NMR (CDCl_3_) and ^13^C-NMR (CDCl_3_) data, see Table 1.

*1-Hydroxy-2-methoxy-6-methyl-9,10-anthraquinone* (**2**). Red needles (CH_2_Cl_2_); m.p. 220-221 ºC. UV λ_max_ (EtOH): 421, 278, 262 nm; IR ν_max_ (KBr): 3,467, 1,653, 1,637 cm^-1^; HREIMS 269.0867 [M + H]^+^ (calc for C_16_H_12_O_4_ 268.2796); For ^1^H-NMR (CDCl_3_) and ^13^C-NMR (CDCl_3_) data, see Table 1.

### 3.5. Determination of Antiplasmodial Activity

The antiplasmodial activity of dichloromethane extract and the isolated compounds were determined by methods as previously described [25]. The samples were dissolved in DMSO and kept at -20 °C until used. The malarial parasite *P. falciparum* (3D7) clone was propagated in a 24-well culture plate in the presence of 10, 1, 0.1, 0.01 and 0.001 µg/mL range of concentrations of each compound. Chloroquine diphosphate was used as positive control. The growth of the parasite was monitored by making a blood smear fixed with MeOH and stained with Geimsa (Merck). The antiplasmodial activity of each compound was expressed as an IC_50_ value, defined as the concentration of the compound causing 50% inhibition of parasite growth relative to an untreated control. 

## 4. Conclusions

A phytochemical study on dried roots of *R. elliptica* afforded a new anthraquinone, 1,2-dimethoxy-6-methyl-9,10-anthraquinone (**1**), together with ten known anthraquinones **2**-**11**. Several anthraquinones strongly inhibited *in vitro* growth of a chloroquine sensitive strain of *Plasmodium falciparum* (3D7), with the strongest inhibition shown by compound **7**. The antiplasmodial data suggested that the roots extract of *R. elliptica* is a potential source of new antiplasmodial agents.
